# Ozone Eliminates SARS-CoV-2 from Difficult-to-Clean Office Supplies and Clinical Equipment

**DOI:** 10.3390/ijerph19148672

**Published:** 2022-07-16

**Authors:** Laura B. Torres-Mata, Omar García-Pérez, Francisco Rodríguez-Esparragón, Angeles Blanco, Jesús Villar, Fernando Ruiz-Apodaca, José L. Martín-Barrasa, Jesús M. González-Martín, Pedro Serrano-Aguilar, José E. Piñero, Elizabeth Córdoba-Lanús, Jacob Lorenzo-Morales, Bernardino Clavo

**Affiliations:** 1 Research Unit, Hospital Universitario Dr. Negrín, 35019 Las Palmas de Gran Canaria, Spain; lbtm1002@gmail.com (L.B.T.-M.); frodesp@gobiernodecanarias.org (F.R.-E.); jesus.villar54@gmail.com (J.V.); jmarbars@gobiernodecanarias.org (J.L.M.-B.); josu.estadistica@gmail.com (J.M.G.-M.); 2 Fundación Canaria del Instituto de Investigación Sanitaria de Canarias (FIISC), 35019 Las Palmas de Gran Canaria, Spain; 3 BioPharm Group, Instituto Universitario de Investigaciones Biomédicas y Sanitarias (IUIBS), Universidad de Las Palmas de Gran Canaria, 35016 Las Palmas de Gran Canaria, Spain; 4 Chemical Engineering & Materials Department, Universidad Complutense, 28040 Madrid, Spain; ablanco@ucm.es; 5 Instituto Universitario de Enfermedades Tropicales y Salud Pública de Canarias, Universidad de La Laguna, Tenerife, 38200 La Laguna, Spain; omargp6@gmail.com (O.G.-P.); jpinero@ull.edu.es (J.E.P.); acordoba@ull.edu.es (E.C.-L.); 6 Departamento de Medicina Interna, Dermatología y Psiquiatría, Universidad de La Laguna, Tenerife, 38200 La Laguna, Spain; 7 Red Cooperativa de Enfermedades Tropicales (RICET), Instituto de Salud Carlos III, 28029 Madrid, Spain; 8 CIBER de Enfermedades Infecciosas, Instituto de Salud Carlos III, 28029 Madrid, Spain; 9 CIBER de Enfermedades Respiratorias, Instituto de Salud Carlos III, 28029 Madrid, Spain; 10 Li Ka Shing Knowledge Institute at St Michael’s Hospital, Toronto, ON M5B 1T8, Canada; 11 Lighting Dynamic Technology, SL, 35001 Las Palmas, Spain; fernando.ruiz@hispalux.com; 12 Aquaculture and Wild Species Health, Infectious Diseases, Universitary Institute of Animal Health and Food Safety (IUSA), Universidad de Las Palmas de Gran Canaria, 35413 Arucas, Spain; 13 Red de Investigación en Cronicidad, Atención Primaria y Promoción de la Salud (RICAPPS), Instituto de Salud Carlos III, 28029 Madrid, Spain; pseragu@gobiernodecanarias.org; 14 Servicio de Evaluación y Planificación del Servicio Canario de Salud (SESCS), 38109 Santa Cruz de Tenerife, Spain; 15 Red de Agencias de Evaluación de Tecnologías Sanitarias y Prestaciones del Sistema Nacional de Salud (RedETS), 28071 Madrid, Spain; 16 Departamento de Obstetricia, Ginecología, Pediatría, Medicina Preventiva y Salud Pública, Toxicología, Medicina Legal y Forense y Parasitología, Universidad de La Laguna, Tenerife, 38200 La Laguna, Spain; 17 Chronic Pain Unit, Hospital Universitario Dr. Negrín, 35019 Las Palmas de Gran Canaria, Spain; 18 Radiation Oncology Department, Hospital Universitario Dr. Negrín, 35019 Las Palmas de Gran Canaria, Spain

**Keywords:** COVID-19, SARS-CoV-2, surface disinfection, clinical equipment, office supplies, ozone

## Abstract

(1) Background: Severe acute respiratory syndrome coronavirus type 2 (SARS-CoV-2) continues to cause profound health, economic, and social problems worldwide. The management and disinfection of materials used daily in health centers and common working environments have prompted concerns about the control of coronavirus disease 2019 (COVID-19) infection risk. Ozone is a powerful oxidizing agent that has been widely used in disinfection processes for decades. The aim of this study was to assess the optimal conditions of ozone treatment for the elimination of heat-inactivated SARS-CoV-2 from office supplies (personal computer monitors, keyboards, and computer mice) and clinical equipment (continuous positive airway pressure tubes and personal protective equipment) that are difficult to clean. (2) Methods: The office supplies and clinical equipment were contaminated in an area of 1 cm^2^ with 1 × 10^4^ viral units of a heat-inactivated SARS-CoV-2 strain, then treated with ozone using two different ozone devices: a specifically designed ozonation chamber (for low–medium ozone concentrations over large volumes) and a clinical ozone generator (for high ozone concentrations over small volumes). SARS-CoV-2 gene detection was carried out using quantitative real-time polymerase chain reaction (RT-qPCR). (3) Results: At high ozone concentrations over small surfaces, the ozone eliminated SARS-CoV-2 RNA in short time periods—i.e., 10 min (at 4000 ppm) or less. The optimum ozone concentration over large volumes was 90 ppm for 120 min in ambient conditions (24 °C and 60–75% relative humidity). (4) Conclusions: This study showed that the appropriate ozone concentration and exposure time eliminated heat-inactivated SARS-CoV-2 RNA from the surfaces of different widely used clinical and office supplies, decreasing their risk of transmission, and improving their reutilization. Ozone may provide an additional tool to control the spread of the COVID-19 pandemic.

## 1. Introduction

The World Health Organization (WHO) declared severe acute respiratory syndrome coronavirus type 2 (SARS-CoV-2) a pandemic on 11 March 2020, and almost two years later, it is still causing profound health, economic, and social problems worldwide [1]. The persistence of SARS-CoV-2 on environmental surfaces has been considered a potentially critical factor for viral spread, despite conflicting reports regarding the maintenance of SARS-CoV-2 infectivity on different surfaces [2,3,4,5].

SARS-CoV-2 belongs to the *Betacoronavirus* family and the group IV *Nidovirals* order (Baltimore classification). This group includes positive monocatenary RNA viruses. Its genome contains 80% similarity to SARS-CoV-1; both have a capsid, which confers sensitivity to heating, detergents, and solvents. The survival of SARS-CoV-2 is dependent on the environmental temperature, relative humidity, and pH, as well as the reactivity of the surface where is located. It is viable on unanimated surfaces for prolonged periods of time between 2 h and nine days, and is spontaneously inactivated on copper surfaces between 4h and 8 h, at 24 h on cardboard, at 48 h on stainless steel, and at 72 h on plastics [3,4,6,7]. Recently, our group reported extended survival times of five to seven days, assessed by evaluation of its cytopathic effect in VERO cells (kidney epithelial cells extracted from an African green monkey), and by gene detection in face masks up to 30 days after contamination [5]. Although vaccinations are the top priority for reducing the spread of coronavirus disease 2019 (COVID-19), according to the WHO guidelines the effective prevention and control of infection includes a practical, evidence-based approach to resist disease spreading [8]. Therefore, the effective disinfection of clinical and public materials may play a crucial role in limiting the viral spread and accelerating the reuse of these materials. Within this context, ozone has been a subject of growing interest [9,10]

Ozone (O_3_) is a molecular gas with three oxygen atoms bonded by high-energy covalent bonds, which makes ozone a powerful oxidizing agent and, therefore, a highly antimicrobial agent. Although it is mainly used for water treatment, it has also been proven to be highly effective at eliminating bacteria, fungi, and molds, and inactivating viruses, including the SARS virus, on surfaces and in aerosols suspended in the air [11,12,13,14,15]. Its efficiency depends on the treatment conditions (e.g., concentration, exposure time, temperature, and humidity) and material properties (e.g., surface reactivity and porosity) [7,10,11,16,17,18]. Its high oxidant activity affects polyunsaturated acids in the biological membranes of bacteria, molds, fungi, and viruses, while also oxidizing nucleic acids (DNA and RNA) [19,20]. In addition to its wide microbicide spectrum activity, ozone does not generate reaction by-products due to its rapid decomposition into oxygen in the atmosphere (see equation in Supplemental Material for further details) [21].

The European Union has included ozone as an effective biocide for water waste cleansing (EU Biocidal Products Regulation Nº 528/2012). Due to the physical state of ozone, it tends to expand and occupy the entire volume in which it is contained, which is an advantage when compared to other disinfection systems, such as ultraviolet (UV) irradiation or hypochlorite dissolutions [17,22,23,24].

Ozone can be generated by a diverse set of devices that use electrical corona discharge to produce ozone from oxygen in the air, or that use medical-grade oxygen. In recent years, there has been practically no innovation in the development of ozone generators in industry, due to the low demand for these devices. However, due to the COVID-19 pandemic, the variety, applicability, and versatility of ozone generators are growing quickly [14,15,25,26,27].

Currently, multiple ongoing studies are attempting to optimize the inactivation and/or elimination of SARS-CoV-2 using ozone [10,11,14,15]. It has been reported that ozone treatment is a widely accessible and effective method for the disinfection of several materials from SARS-CoV-2, including personal protective equipment (PPE) for healthcare workers and patients [10,22,25], creating the possibility of safely re-using these clinical and working materials and contributing to their more sustainable use. However, further studies are still necessary to validate these results and facilitate the wide acceptance of this treatment and its inclusion in the list of viable treatments.

In our previous work, we reported the RNA degradation of heat-inactivated SARS-CoV-2 on PPE and masks at high and low ozone concentrations [10]. The aim of the current work was to validate these preliminary results with further materials. Thus, this study was extended to the contaminated surfaces of different office supplies and clinical equipment that are difficult to clean, where the effects of ozone treatment were evaluated for eliminating heat-inactivated SARS-CoV-2 RNA.

## 2. Methods

### 2.1. Samples, Study Design, and Outcome Assessment

Samples of several materials and sizes were contaminated with heat-inactivated SARS-CoV-2. The size of the samples varied from bands of 2 cm × 1 cm (for face masks, and vinyl and nitrile lab gloves) to the entire object in the case of office, clinical, and laboratory supplies (up to 40 cm × 40 cm × 20 cm), including two operative cellphones, inoperative computer accessories (mouse, keyboard, and computer screen), reactant flasks, test tubes, grids, continuous positive airway pressure (CPAP) tubes (100 cm length × 2 cm diameter), and syringe and needle covers (paper tissue).

All the supplies assessed were contaminated with the SARS-CoV-2 strain 2019-nCoV/USA-WA1/2020, which was inactivated by heating at 65 °C for 30 min (ATCC^®^ VR-1986HK™, ATCC, Manassas, VA, USA) at 1 × 10^3^ copies/µL. In all cases, the volume of the contamination drop was 10 µL (occupying a surface equivalent to 1 cm^2^), corresponding to 1 × 10^4^ copies, which was considered a reasonable amount of virus to remain stable on a surface for enough time to experimentally evaluate the virucidal activity of the procedure [7,13,28]. After that, the samples were allowed to dry in a laminar flow hood until treatment with the corresponding ozone conditions.

For the first assay, we used low concentrations of ozone (19 ppm) under high relative humidity (80–95%) to reinforce the ozone effect at low concentrations, as previously reported [7,10,11,28,29,30]. In the following eight assays, the temperature (21.8–24.7 °C) and relative humidity (60–75%) were those of room conditions, controlled by the integral air conditioning system of the hospital.

For each study performed on every supply type, two samples were used for the control (confirmation “pre-treatment” column) and another two samples were used for the O_3_ treatment (“post-treatment” column).

The primary outcome measure was the detection yield of heat-inactivated SARS-CoV-2 gene amplification after ozone treatment, as assessed by quantitative real-time polymerase chain reaction (RT-qPCR).

Table 1 shows the different materials and supplies assessed in the different ozone treatment conditions.

### 2.2. Ozone Exposure Conditions

The ozone treatment procedures were performed at the Hospital Universitario de Gran Canaria Dr. Negrín (Las Palmas de Gran Canaria, Spain).

For the high-concentration assays, the ozone was produced using a medical ozone generator (Ozonobaric P^®^, Sedecal, Madrid, Spain). This device generates ozone from medical-grade oxygen, obtaining an O_3_/O_2_ gas mixture between 500 ppm and 40,000 ppm (1–80 g/m^3^) in relatively small volumes. During the procedure, the samples contaminated with heat-inactivated SARS-CoV-2 were introduced one at a time in a 60 mL syringe (when possible) or inside a plastic bag. Then, the air was expelled by a vacuum. Later, an O_3_/O_2_ gas mixture was introduced at selected concentrations of 2000, 4000, or 10,000 ppm (4, 8, or 20 g/m^3^, respectively) with short exposition times (5 or 10 min) based on our previous study [10].

The low-concentration assays were performed with a size-adaptable ozonation chamber UVOZ^®^ (designed and performed by Lighting Dynamic Technology, Las Palmas de Gran Canaria, Spain). The chamber was used with a cabinet with dimensions of 200 × 100 × 100 cm^3^ (2000 L) and was equipped with a 65 W industrial ozone generator, which produced ozone from the oxygen present in the environmental air (21%). It was also equipped with a set of two ultraviolet (UV) lamps and a humidifier to be used as required. Using the large volume of the ozonation chamber, we analyzed the effect of low ozone concentrations (19, 33, 70, and 90 ppm = 0.038, 0.066, 0.140, and 0.180 g/m^3^, respectively) for longer exposure times (30, 60, 90, and 120 min). To compensate for the spontaneous decomposition of ozone to oxygen (half-life of 40 min at 20 °C and 25 min at 30 °C), the ozonation chamber’s ozone generator was switched on and off to maintain the concentration at the desired values during the experiments. Table 1 shows the ozone treatment conditions.

For each supply, at every evaluated ozone condition, two units were used. For each unit we obtained two pre-ozone control samples and two post-ozone samples, which were collected with swabs (or by cutting the sample to pick up the drop) and maintained in 3 mL universal transport medium (UTM-RT™, COPAN Diagnostics, CA) for conservation and further PCR analyses. Each sample was assessed in duplicate by RT-qPCR for the amplification of SARS-CoV-2 genes.

### 2.3. Quantitative Real-Time Polymerase Chain Reaction (RT-qPCR)

Detection of viral RNA by RT-qPCR was performed at the Instituto Universitario de Enfermedades Tropicales y Salud Pública de Canarias (La Laguna, Tenerife, Spain) according to the WHO guidelines for Biosafety Level 2 facilities [31]. The previously described samples were processed to extract viral RNA using a Maxwell 16S Viral RNA Mini Kit (Promega, Madrid, Spain) following the manufacturer’s recommendations. The extracted RNA was resuspended in 50 µL of elution buffer and used for RT-qPCR.

For viral gene detection by RT-qPCR, the TaqPath™ COVID-19 CE-IVD RT-qPCR Kit (Applied Biosystems, Thermo Fisher Scientific, Madrid, Spain) was used, following the manufacturer’s instructions. This kit included assays targeting three SARS-CoV-2 genes (Gene ORF1ab, N Protein, and S Protein), and an MS2 Phage as a control for the RNA extraction. It also contained a positive TaqPath™ COVID-19 control. Each sample was analyzed in duplicate with a QuantStudio 3™ Real-Time qPCR System (Applied Biosystems). All RT-qPCR samples were assessed in duplicate. Positive results were considered when amplification genes had Ct values <37 (Ct: Cycle threshold related to the number of cycles required for the fluorescently marked amplification to cross the threshold in the RT-qPCR reaction).

## 3. Results

### 3.1. Low Ozone Concentrations

At 19 ppm (0.038 g/m^3^) and controlled relative humidity (80–90%), viral gene amplification was detected in face masks after 30 min (two genes) and 60 min (only one gene) of ozone treatment. For the vinyl lab gloves, no amplification was observed after 30 min of treatment.

At 33 ppm (0.066 g/m^3^) and standard relative humidity, only the office supplies were studied, and viral genes were detected in all the analyzed materials (computer mouse, computer screen, and keyboard keys).

At 70 ppm (0.140 g/m^3^) and standard relative humidity, the clinical equipment, and office supplies were studied. The treatment was effective for six of the nine materials, including the lab grid, reactant flask, test tube, computer screens, keyboard keys, and cellphone screens. In the CPAP tube, the contaminated drop at 50 cm from the entry point of O_3_ showed amplification in only one gene.

At 90 ppm (0.180 g/m^3^) and standard relative humidity, all evaluable clinical equipment and office supplies showed no SARS-CoV-2 gene amplification after ozone treatment.

**Figure 1:**
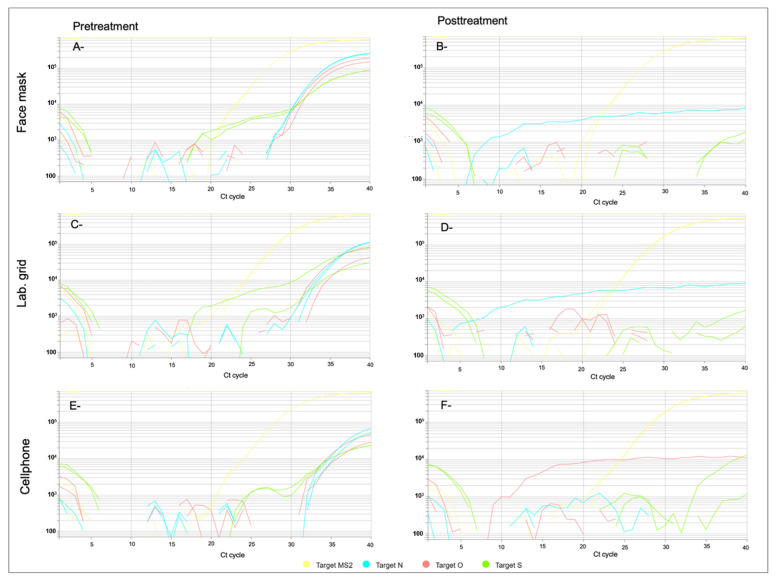
Heat-inactivated SARS-CoV-2 gene evaluation by RT-qPCR for (**A**,**B**) face mask, (**C**,**D**) lab grid, and (**E**,**F**) cellphone treated with 90 ppm of ozone for 120 min (65–70% humidity). Target MS2: MS2 Phage as a control for the RNA extraction; Target O, N, and S: specific SARS-CoV-2 target sequences in the ORF1ab, nucleocapsid, and spike protein gene respectively. (**A**,**C**,**E**) images correspond to materials before exposure to ozone that showed Ct values <37, indicating positive amplification of the three viral genes. (**B**,**D**,**F**) images correspond to materials treated with ozone that showed no amplification of the viral targets O, N, or S, where only the control target MS2 was amplified.

Table 1 shows the results of the different ozone exposure conditions. Figure 1 shows the RT-qPCR results of heat-inactivated SARS CoV-2 genes evaluated for three different materials (face mask, cellphone, and lab grid) treated with ozone at 90 ppm for 120 min at 65–70% relative humidity.

### 3.2. High Ozone Concentrations

At 2000 ppm (4 g/m^3^) and standard relative humidity, we only analyzed face masks, and they did not show gene amplification after 10 min nor 5 min of O_3_ exposition.

At 4000 ppm (8 g/m^3^) and standard relative humidity, there was no gene amplification after 5 min in face masks, and there was no gene amplification after 10 min on the computer mouse, computer screen, keyboard keys, nor between keys of the keyboard. However, one gene remained amplified after 10 min in the contaminated drop at 1 m (100 cm) from the entry point of O_3_.

At 10,000 ppm (20 g/m^3^), and standard relative humidity, there was no viral RNA detection after 10 min in any clinical equipment nor office supplies, including the areas between keys of the keyboard, nor the contaminated drops at 1 m (100 cm) from the entry point of O_3_. Table 1 shows the results obtained under low and high ozone concentrations.

## 4. Discussion

This study indicates that ozone could eliminate heat-inactivated SARS-CoV-2 genes from different contaminated surfaces of several office and clinical supplies. This effect was highly dependent on the ozone concentration, time exposure, and material used. At a standard relative humidity (60–75%) and a temperature of 21.8–24.7 °C, we found that the best disinfection conditions were 90 ppm for 120 min for large volume supplies. However, for smaller volumes, 4000 ppm for 10 min was sufficient, although 10,000 ppm was required for surfaces that were more difficult to access (i.e., 100 cm CPAP tube).

As previously reported, at low ozone concentrations, high-humidity conditions reinforce ozone activity [7,10,11,28,29,30]. Thus, the disinfection treatment was started at an ozone concentration of 19 ppm (0.038 g/m^3^) and 80–95% relative humidity. The samples initially selected were vinyl lab gloves and face masks, due to the high use of these tools during the COVID-19 pandemic. The treatment was applied to the vinyl lab gloves for 30 min, and to the face masks for 30 and 60 min. The treatment was successful for the vinyl lab gloves, which did not show the amplification of SARS-CoV-2 genes, but not for the face masks. The improved results for the gloves compared to those for the face masks could be due to the less porous surface of gloves that may allow ozone to easily access and react effectively with the viral RNA. Although the reported survival time of SARS-CoV-2 on plastic materials is 72 h (polypropylene in masks and polyvinylchloride in gloves), their microscopic interactions seem to influence the efficacy of the procedure [4]. This finding is in agreement with our previous report showing the elimination of heat-inactivated SARS-CoV-2 from PPE gowns at low ozone concentrations (4–6.5 ppm) under 99% relative humidity, but not from face masks [10]. In the current study, we did not use 99% relative humidity, because in previous work we found water condensation on the surfaces inside the ozonation chamber. The 80–90% relative humidity used in the first assay did not lead to water condensation on the surfaces inside the ozonation chamber. Finally, because the study included electronic and personal computer (PC) components, we decided to evaluate the ozone effects under standard relative humidity, which is more tolerable for many electronic devices.

Inside the ozonation chamber after 120 min of ozone exposure, we observed that: (i) at an ozone concentration of 33 ppm (0.066 g/m^3^), SARS-CoV-2 RNA maintained its integrity, as observed by the amplification of viral genes; (ii) at a concentration of 70 ppm (0.140 g/m^3^), ozone was effective on six out of nine samples; and (iii) at a concentration of 90 ppm (0.180 g/m^3^), ozone was effective in eliminating SARS-CoV-2 RNA from all the tested surfaces. This work also assessed the efficacy of the use of very high ozone concentrations with short exposure time for degrading the RNA of SARS-CoV-2. Ozone treatments at 4000 ppm (8 g/m^3^) and 2000 ppm (4 g/m^3^) for five minutes were effective for face masks, in agreement with previous reports [10,32], whilst ozone exposure at 4000 ppm for 10 min was effective for all surfaces, except the 100 cm CPAP tube (where one of the three SARS-CoV-2 genes was detected); no SARS-CoV-2 genes were detected in any sample (100 cm CPAP tube included) after 10 min at 10,000 ppm (20 g/m^3^).

**Figure 2:**
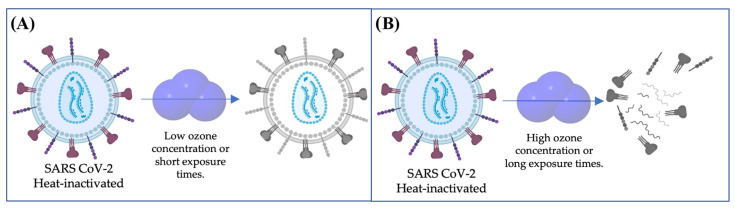
Proposed degradation effects of ozone on the biomolecules of a SARS-CoV-2 viral particle. (**A**) Low ozone concentrations or short exposure times mainly lead to degradation of lipids and proteins but not viral RNA, which is detected by RT-qPCR. (**B**) High ozone concentrations or long exposure times also alter and oxidize the viral RNA, which therefore is not detected by RT-qPCR.

According to previous reports, the ozone levels required to inactivate viral particles are quite low compared to those evaluated in this study for viral RNA elimination [11]. This could be due to the role of envelope integrity in maintaining the infectivity of the viral particle, and the relatively lower reactivity of RNA to oxidation compared to other biomolecules. Lipids, by peroxidation, and proteins of the viral capsid, by losing their tridimensional structure, affect the infectious capacity of the virus [19,27], although RNA can persist and be detected by RT-qPCR (Figure 2). The inactivation of several viruses through protein shell damage and lipid envelope peroxidation by ozone has been previously reported [19,20]. A recent report described three main mechanisms for the elimination of SARS-CoV-2 by ozone: (i) the peroxidation of unsaturated fatty acids, which leads to the disturbance of the viral envelope formation; (ii) unsettling of the amino acid structure, which collaborates with the viral envelope damage and leads to the oxidation of cysteine to cystine; and (iii) the latter alongside the release of Zn^+2^ from the viral non-structural proteins, leading to secondary and tertiary structure alterations in those non-structural proteins [33].

**Figure 3:**
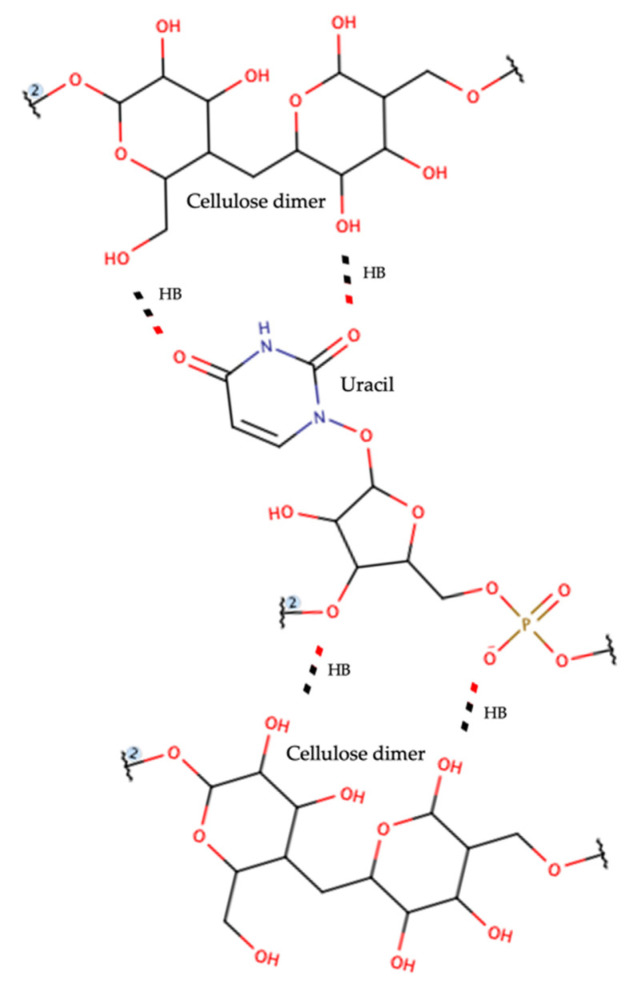
Representation of suggested hydrogen bond interactions between cellulose and RNA. HB, hydrogen bond.

When assessing the paper samples, including syringe covers, reactant flask tags, and needle covers, we observed a particular behavior. We expected gene amplification in all samples before ozone treatment; however, there viral amplification was observed only in the pre-ozone needle cover samples, with no amplification in the pre-ozone syringe cover or reactant flask tag samples. Thus, the absence of amplification in the post-ozone paper samples was not valuable. In previous viability studies of SARS-CoV-1 and SARS-CoV-2 on several materials, the data were reported as “noisier” for experiments using cardboard or cotton (which are made of cellulose), both of which have a high adsorption capacity [3,34,35]. This finding is likely due to the interactions between those biomaterials and the viral particles caused by the intermolecular forces (i.e., hydrogen bonds) between RNA hydroxyl groups and cellulose polar groups. The use of cellulose columns to purify nucleic acids supports this hypothesis [36,37,38]. See Figure 3.

These interactions are thought to lead to the “adsorption” of RNA, which will not be detected by RT-qPCR. The stability and interactions of SARS-CoV-2 RNA with highly polar and porous materials, such as paper and cardboard, should be further studied.

On the other hand, the survival of SARS-CoV-2 on unanimated surfaces has been described as between 4 and 8 h on copper surfaces, 24 h on cardboard, 48 h on stainless steel, and 72 h on plastics [2,3,7]. The long-term survival of SARS-CoV-2 on plastics (a widely used material) is a risk for contamination and propagation among people [35]. Recent data from our group found SARS-CoV-2 RNA on the surface of contaminated face masks after 30 days [5]. Sodium hypochlorite (bleach) is the standard method for cleaning at-risk surfaces. However, not all materials can be treated with this method or with liquids, as is the case for face masks, some PPE, paper or cardboard packaging (e.g., syringe covers, needle covers, reactant flask tags, etc.), electronic devices, and some potentially reusable materials such as CPAP tubes.

Our findings support the effectiveness of ozone treatment for degrading SARS-CoV-2 RNA on the surface of several difficult-to-clean supplies from clinical and office environments that cannot be thoroughly cleaned using sodium hypochlorite. The treatment of these supplies with ozone could represent a safe approach to prevent or decrease the risk of contamination with SARS-CoV-2 in hospitals, nursing homes, and more general environments. The use of ozonation chambers can facilitate the simultaneous treatment of large or multiple devices and supplies, potentially including full PPE, instead of using lower ozone concentrations with longer exposure times. Furthermore, the treatments can be performed in small rooms. If faster disinfection is required (for example, for fast reutilization), smaller volumes with higher ozone concentrations from clinical ozone devices could be used for 5–10 min. Both procedures were completely safe for the operators. A n operational strength of the ozonation chambers is the versatility of their application. They allow treatment of different types and sizes of materials and the modulation of relative humidity.

Our results support the potential use of ozone for the re-utilization of certain materials under conditions of very low availability. Additionally, ozone does not generate contaminating decomposition subproducts (O_3_ spontaneously degrades to O_2_), which could decrease biological risk in the management or elimination of hazardous materials. This can facilitate the re-utilization of supplies, decreasing waste materials, which is in line with the green chemistry technologies associated with the Green Deal Goal of the European Union: preserving our environment [39].

We acknowledge some limitations of our study. First, the analyses of the computer mouse and keyboard samples and the paper surface samples (reactant flask tag and syringe cover) were not evaluable, because the pre-ozone control samples did not show viral gene amplification. After the planned analysis of two units for each supply and two samples for each unit produced the same results, we decided not to perform further studies. Second, nitrile and latex react very easily with ozone and were excluded from ozone treatment [40]. Third, the PC components were inoperative when treated, and it was not possible to evaluate the potential adverse effect of ozone on their functionality. However, working cellphones remained operative after ozone treatment, and the quality of the materials was not macroscopically affected. Further evaluation is required of the effects on these materials of chronic exposure to ozone, especially electronic components.

## 5. Conclusions

This study shows that an appropriate ozone concentration and exposure time can eliminate heat-inactivated SARS-CoV-2 RNA from the surfaces of different widely used clinical and office supplies, decreasing their management risk and improving their reutilization. Our findings support that ozone could provide an additional tool to control the spread of the COVID-19 pandemic. The optimal treatment conditions (concentration and time of ozone exposure) varied according to the composition and volume of the different materials. Further research is required into the effects of the chronic exposure of these materials to ozone, especially electronic components. The final real value of the procedure could also depend on the variable costs and availability of the different materials to be treated. It is necessary to develop new knowledge before extending the use of ozone in this context.

## 6. Patents

The initial ozonation chamber UVOZ^®^ and a further update used in the study have been patented (No. U202030703 and No. P202130273, respectively) by Lighting Dynamic Technology, S.L., with the participation of 10 of the authors of this manuscript: F.R.A., B.C., E.C.-L., F.R.-E., J.E.P., J.V., A.B., J.L.M.-B., J.M.G.-M., P.S.-A., and J.L.-M.

## Figures and Tables

**Table 1:** SARS-CoV-2 gene amplification by quantitative real-time polymerase chain reaction (RT-qPCR) in clinical and office supplies contaminated by a heat-inactivated strain, after treatment with different ozone exposure conditions (concentration, time, and relative humidity).

Supply Type (by Duplicate)	Ozone Concentration (ppm)	Time of Treatment (Minutes)	Relative Humidity (%)	RT-qPCR	
				Pre-Treatment	Post-Treatment
Face masks	19	30	80–90	✓✓✓	✓✓
	19	60	85–90	✓✓✓	✓
	90	120	65–70	✓✓✓	X
	2000	5	60–75	✓✓✓	X
	2000	10	60–75	✓✓✓	X
	4000	5	60–75	✓✓✓	X
Vinyl lab glove	19	30	80–90	✓✓✓	X
Nitrile lab glove	90	120	65–70	✓✓✓	X
Cover needle	90	120	65–70	✓✓✓	X
Cover syringe	90	120	65–70	X	n.v.
CPAP tube	70 ^a^	120	60–75	✓✓✓	✓
	90 ^a^	120	65–70	✓✓✓	X
	4000 ^b^	10	60–75	✓✓✓	✓
	10,000 ^b^	10	60–75	✓✓✓	X
Lab grid	70	120	60–75	✓✓✓	X
	90	120	65–70	✓✓✓	X
Reactant flask	70	120	60–75	✓✓✓	X
	90	120	65–70	✓✓✓	X
Reactant flask tag	70	120	60–75	X	n.v.
	90	120	65–70	X	n.v.
Test tube	70	120	60–75	✓✓✓	X
	90	120	65–70	✓✓✓	X
Between keys of mouse	70	120	60–75	X	n.v.
	90	120	65–70	X	n.v.
Computer mouse	33	120	60–75	✓✓✓	✓
	70	120	60–75	✓✓✓	✓✓✓
	90	120	65–70	✓✓✓	X
	4000	10	60–75	✓	X
	10,000	10	60–75	✓	X
Computer screen	33	120	60–75	✓✓✓	✓✓✓
	70	120	60–75	✓✓✓	X
	90	120	65–70	✓✓✓	X
	4000	10	60–75	✓✓✓	X
	10,000	10	60–75	✓✓✓	X
Keyboard key	33	120	60–75	✓✓✓	✓✓✓
	70	120	60–75	✓✓✓	X
	90	120	65–70	✓✓✓	X
	4000	10	60–75	X	n.v.
	10,000	10	60–75	X	n.v.
Between keys of keyboard	33	120	60–75	✓✓✓	✓✓✓
	70	120	60–75	✓✓✓	✓✓✓
	90	120	65–70	X	n.v.
	4000	10	60–75	✓✓✓	X
	10,000	10	60–75	✓✓✓	X
Cellphone	70	120	60–75	✓✓✓	X
	90	120	65–70	✓✓✓	X

For each study performed on every supply type, two samples were used for the control (confirmation “pre-treatment” column) and another two samples were used for the O_3_ treatment (“post-treatment” column). For each sample, RT-qPCR was performed in duplicate. X, no amplification; ✓, one positive gene; ✓✓, two positive genes; ✓✓✓, three positive genes; CPAP, continuous positive airway pressure; n.v., not valuable due to negative result in the control in the pretreatment group; ^a^ contaminating drop at 50 cm from the entry point of O_3_; ^b^ contaminating drop at 1 m (100 cm) from the entry point of O_3_.

## Data Availability

Not applicable.

## Supplementary Materials

The following supporting information can be downloaded at: https://www.mdpi.com/article/10.3390/ijerph19148672/s1, Equation (S1): The destruction of ozone in the atmosphere.

## Abbreviations

COVID-19	Coronavirus Disease 2019
CPAP	Continuous positive airway pressure tube
DNA	Deoxyribonucleic acid
O_2_	Molecular oxygen
O_3_	Ozone
PPE	Personal protective equipment
RNA	Ribonucleic acid
RT-qPCR	Real-time polymerase chain reaction
SARS-CoV-2	Severe acute respiratory syndrome coronavirus 2
UV	Ultraviolet
VERO	African green monkey kidney epithelial cells
WHO	World Health Organization
