# Supplementary figures and images for: Ozone Eliminates SARS-CoV-2 from Difficult-to-Clean Office Supplies and Clinical Equipment
Source: Int J Environ Res Public Health. 2022 Jul 16;19(14):8672. doi: 10.3390/ijerph19148672 (PMC9321385; doi:10.3390/ijerph19148672)

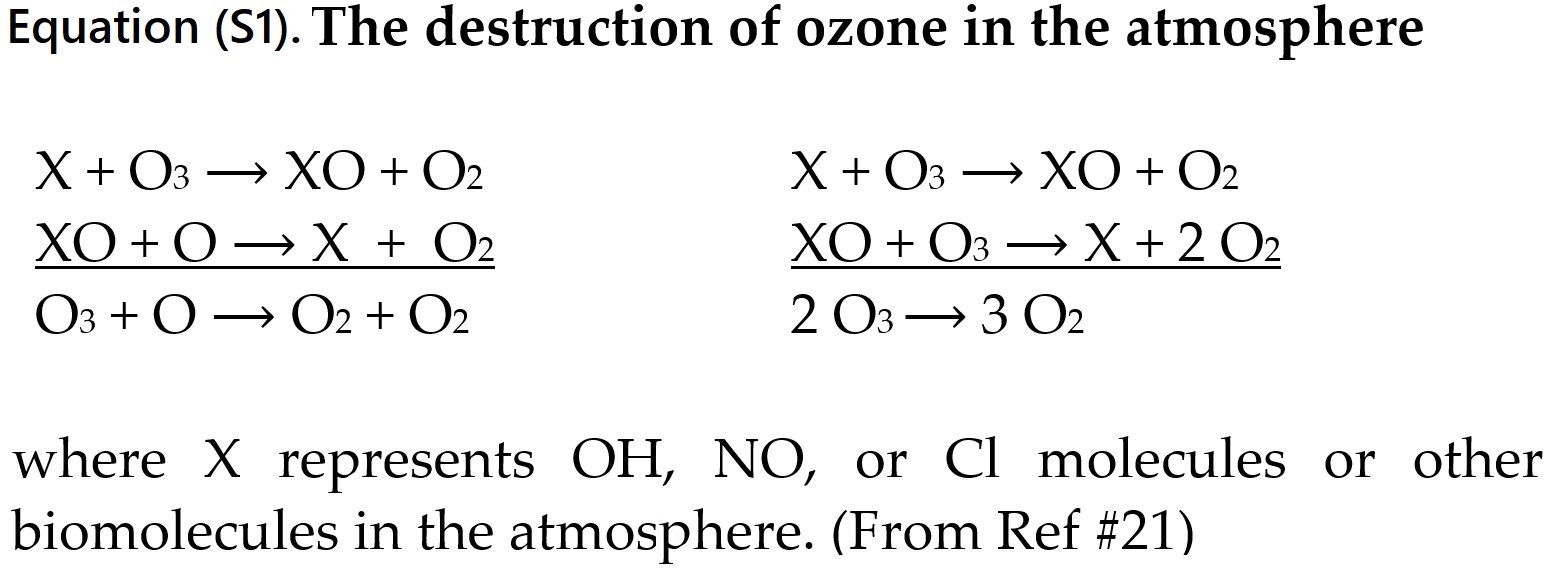

Supplement: Supplementary file 1 [file ijerph-19-08672-s001.zip › ijerph-1586901-supplementary.jpg]
